# High serum mannose in colorectal cancer: a novel biomarker of lymph node metastasis and poor prognosis

**DOI:** 10.3389/fonc.2023.1213952

**Published:** 2023-08-22

**Authors:** Xueling Wang, Haoran Li, Xiaotian Chang, Zibin Tian

**Affiliations:** ^1^Center for Clinical Research, The Affiliated Hospital of Qingdao University, Qingdao, China; ^2^Department of Gastroenterology, The Affiliated Hospital of Qingdao University, Qingdao, China; ^3^Department of Hepatobiliary and Pancreatic Surgery, The Affiliated Hospital of Qingdao University, Qingdao, China

**Keywords:** colorectal cancer, mannose, lymph node metastasis, prognosis, biomarker

## Abstract

**Background:**

Lymph node status is an important prognostic indicator and it significantly influences treatment decisions for colorectal cancer (CRC). The objective of this study was to evaluate the ability of serum monosaccharides in predicting lymph node metastasis (LNM) and prognosis.

**Methods:**

High performance anion exchange chromatography coupled with pulsed amperometric detector (HPAEC-PAD) was used to quantify serum monosaccharides from 252 CRC patients. Receiver operating characteristic (ROC) curves were used to evaluate predictive performance of parameters. Predictors of LNM were evaluated by univariate and multivariate analyses. The prognostic role of the factors was evaluated by survival analysis.

**Results:**

The levels of serum mannose (Man) and galactose (Gal) were significantly increased in patients with LNM (*p <*0.0001, *p* =0.0017, respectively). The area under the curves (AUCs) of Man was 0.8140, which was higher than carcinoembryonic antigen (CEA) (AUC =0.6523). Univariate and multivariate analyses demonstrated histologic grade (G3) (odds ratio [OR] =2.60, *p* =0.043), histologic grade (mucin-producing subtype) (odds ratio [OR] =3.38, *p* =0.032), lymphovascular invasion (LVI) (OR =2.42, *p <*0.01), CEA (>5ng/ml) (OR =1.85, *p* =0.042) and high Man (OR =2.65, *p* =0.006) to be independent risk factors of LNM. The survival analysis showed that the high serum Man was independent risk factor for poor prognosis in CRC patients (HR=1.75, *p* =0.004).

**Conclusions:**

The Man is superior to CEA in prediction of LNM for CRC patients. Man is expected to be a predictor for LNM in CRC. High serum Man is associated with poor prognosis of CRC patients.

## Introduction

The morbidity and mortality rates of CRC have been rising rapidly over the last decade. There were 1.33 million new cases and 694 thousand CRC-caused deaths in 2012, and the numbers had respectively risen to 1.88 million and 916 thousand by 2020 (1–3). In clinical practice, lymph node metastasis (LNM) is associated with poor prognosis, and it also influences treatment decisions (4). For instance, preoperative assessment of the likelihood of LNM could be used as a basis to advise neoadjuvant chemotherapy in CRC patients (4). In addition, in patients with early CRC (cT1), endoscopic therapy is feasible only when the possibility of LNM is negligible (5). However, up to now, it is still not accurate enough to predict LNM preoperatively (4). Some studies have postulated lymphovascular invasion (LVI) and poorly differentiated components as suggestive predictors of LNM (6, 7). However, these pathological data are generally not available until surgery. Besides these factors, biomarkers have also been investigated, and the combination of biomarkers and pathological parameters may increase the accuracy of LNM prediction (7).

The complexity of polysaccharide or glycan structures arises not only from non-template biosynthesis but also from different monosaccharides with multiple linkage positions (8). In fact, all human glycans consist of nine monosaccharides, including mannose (Man), sialic acid (SA), fucose (Fuc), xylose (Xyl), galactose (Gal), glucose (Glc), galactosamine (GalN), glucosamine (GlcN) and glucuronic acid (GlcA) (9). Glycan synthesis is the most complex post-translational modification of proteins. The various glycan structures significantly influenced biological functions of glycoprotein (10). Morever, glycans located on the surface of cells were actively involved in cellular events and they could impact the properties and behavior of cells (11). In previous study, alterations of glycosylation have been found in various cancers (12). Structural alteration in glycans has been recognized as biomarkers, which could be used in the tumor diagnosis, LNM prediction and prognosis assessment (13, 14). Although the tremendous ability of glycans on modulating glycoprotein function has long been recognized, the complexity of glycan structures and the diversity of glycosylation combinations have prevented the progress of glycan research (15). In response, our research group developed a method to obtain the composition of serum monosaccharides. The method could detect six monosaccharides at once, including Glc, Fuc, GlcN, GalN, Gal, and Man (8). In this study, we analyzed the relationship between serum monosaccharide levels and lymph node status. In addition, we analyzed the predictors of LNM in CRC patients. Further, based on the follow-up data, we assessed the risk factors for poor prognosis.

## Materials and methods

### Serum sample collection

**Figure 1:**
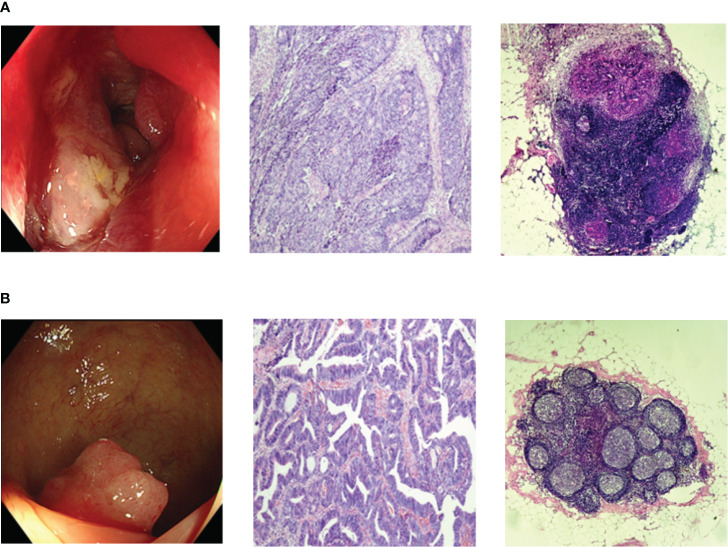
Representative endoscopic and histopathologic images. **(A)** Endoscopic and histopathologic images of LNM-positive CRC patients; **(B)** Endoscopic and histopathologic images of LNM-negative CRC patients;. CRC, colorectal cancer; LNM, lymph node metastasis.

From January 2019 to June 2020, a total of 252 fasting blood samples were collected. The serum samples were collected from CRC patients by laboratory physician in our hospital according to standardized procedure and doctor’s prescriptions. There were 122 LNM-positive patients and 130 LNM-negative patients. The diagnosis of CRC and LNM were confirmed by endoscopy and postoperative pathology, as shown in **Figure 1**. Patients with history of prior tumor, radiotherapy, chemotherapy and type 2 diabetes mellitus (T2DM) were excluded. This study was designed on the basis of the Declaration of Helsinki of the World Medical Association. The research protocol was approved by the Ethics Committee of the Affiliated Hospital of Qingdao University (QYFYWZLL27534). And all the patients had signed informed consents.

### Serum monosaccharides detection

The serum monosaccharide composition was analyzed as described in the previous report (8). In short, 2uL serum, 8uL deionized water and 10uL (6mol/L) HCl solution were added into the microwave degradation tube successively. Then the mixtures were hydrolysed in a microwave reactor (CEM, Germany) for 10min. The HCl was then removed by centrifugal drying (LABCONCO, Germany). Each pellet was dissolved in 150μL deionized water and then collected supernatant after centrifuging at 13,000 r/min for 10 min. Finally, the serum monosaccharides including Man, Fuc, Gal, GalN, Glc and GlcN were detected by high performance anion exchange chromatography coupled with pulsed amperometric detector (HPAEC-PAD) (Thermo, USA).

### Data collection

According to the Japanese Society for Cancer of the Colon and Rectum (JSCCR) treatment guidelines, the included patients later underwent colon/rectal resection with lymph node dissection, and the retrieved histologic slides were examined by two experienced pathologists individually.

Based on the most predominant histologic feature, tumors were classified as well, moderately, and poorly differentiated adenocarcinomas or signet ring cell type or mucinous carcinoma (6). According to American Joint Committee on Cancer (AJCC) TNM staging classification and Union for International Cancer Control (UICC), tumor invasion depth was divided into the following four grades: T1(tumor invasion did not exceed the submucosa), T2 (tumor invasion into muscularis propria), T3(invasion depth reached subserosa), and T4(tumor invasion into the viscera peritoneum or adjacent structures or organs). D2-40 antibody was used to identified LVI (Dako, Denmark). Perineural invasion was diagnosed by detecting S100 protein.

Clinical and histopathological data of all patients were collected, including sex, age, body mass index (BMI), carcinoembryonic antigen (CEA) level, major tumor size, tumor location, histologic grade, depth of invasion, perineural invasion, LVI, and lymph node status.

### Statistical analysis

All data were analyzed with SPSS statistical software (version 22.0) and GraphPad Prism 8.3. 0. T test was used to analyze quantitative data with normal distribution, while Mann-Whitney U test analyzed those without normal distribution. One-way ANOVA or Kruskal-Wallis test was used to analyze quantitative variables with three or more groups. Chi-square test or fisher exact test was used for univariate analysis. The significant variables in the univariate analysis were subsequently entered into a multivariate logistic regression analysis to acquire the independent risk factors for LNM. Log-rank test was performed for survival analysis. Prognostic factors were drawn from univariate and multivariate Cox proportional hazards models. Spearman correlation analysis assessed the relationship between the CEA and differentially expressed monosaccharides. Receiver operating characteristic (ROC) curves were used to evaluate the predictive performance of makers. All reported *p*-values were double-tailed, and *p*-value <0.05 was considered statistically significant.

## Results

### The expression of serum monosaccharides according to LNM status

**Figure 2:**
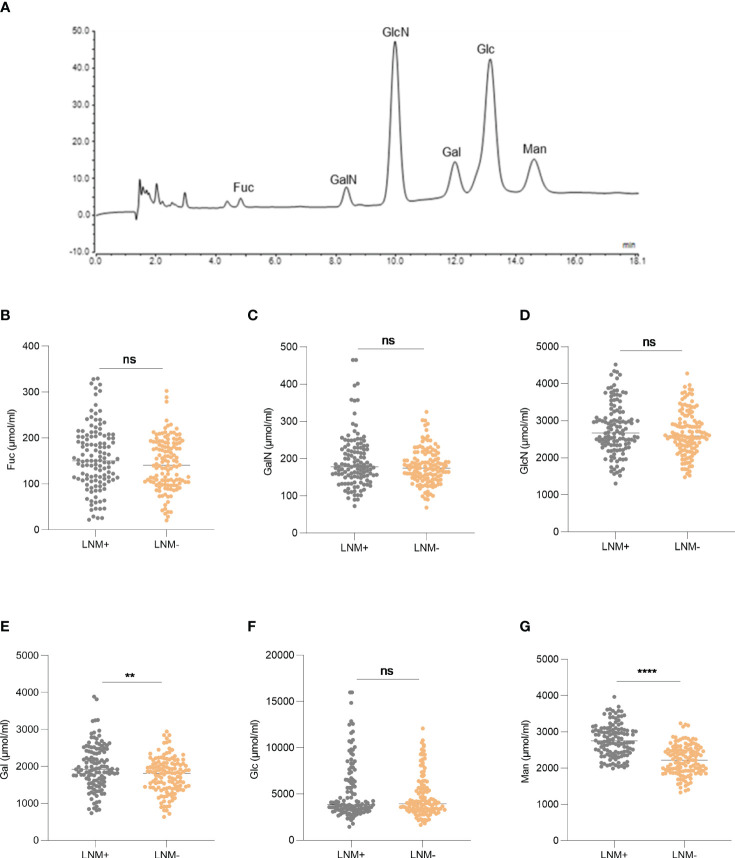
The expression of serum monosaccharides according to LNM status. **(A)** HPAEC-PAD chromatogram of monosaccharides. The expression of Fuc **(B)**, GalN **(C)**, GlcN **(D)**, Gal **(E)**, Glc **(F)**, Man **(G)** according to LNM status. LNM+: patients with lymph node metastasis; LNM-: patients without lymph node metastasis. LNM, lymph node metastasis; Fuc, fucose; GalN, galactosamine; GlcN, glucosamine; Gal, galactose; Glc, glucose; Man, mannose. ns, not significant, **p ≤0.01, ****p ≤0. 0001.

A total of 252 serum samples were collected, including 122 LNM-positive patients and 130 LNM-negative patients. The optimized MAAH plus HPAEC-PAD method (8) was used to detect the levels of six hydrolyzed monosaccharides in the sera of CRC patients. The chromatogram of monosaccharides was shown in **Figure 2A**. Levels of Gal and Man increased significantly in CRC patients with LNM (*p* =0.0017, *p <*0.0001, respectively). However, there was no significant difference in levels of Fuc, GalN, GlcN and Glc between patients with LNM and those without LNM (**Figures 2B–G**).

**Figure 3:**
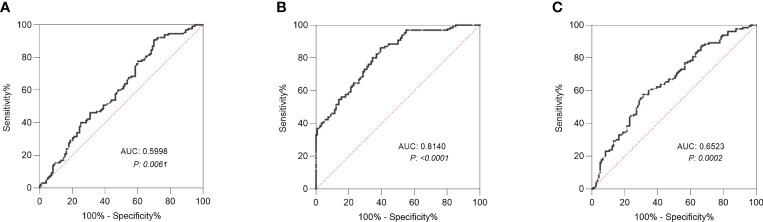
Receiver operating characteristic (ROC) curve analysis. **(A)** ROC curve analysis of Gal was conducted to differentiate CRC patients with LNM from those without LNM. **(B)** ROC curve analysis of Man was conducted to differentiate CRC patients with LNM from those without LNM. **(C)** ROC curve analysis of CEA was conducted to differentiate CRC patients with LNM from those without LNM. LNM, lymph node metastasis; Gal, galactose; Man, mannose. AUC, area under the curve; CEA, carcinoembryonic antigen.

ROC curve was used to evaluate the ability of factors to predict LNM. The area under the curve (AUC) of Gal was 0.5998 with a cut-off value of 2346 µmol/mL (**Figure 3A**). And the AUC of Man was 0.8140 with a cut-off value of 2663 µmol/ml (**Figure 3B**), whereas the AUC of CEA was 0.6523 (**Figure 3C**).

### Clinicopathologic features of enrolled CRC patients

The prevalence of LNM in patients with tumor size≥2cm was 55.56% (52/90), which was higher than in patients with tumor size<2cm (43.21%, 70/162) (*p* =0.027). Regarding histologic grade, LNM occurred more frequently in patients with G3 (*p* =0.002) and mucin-producing subtype (*p <*0.001) than in those with G1 and G2. Regarding depth of invasion, 33.33% (16/48) of the T1/T2 patients were LNM-positive, while 51.96% (106/204) of the T3/T4 patients were LNM-positive (*p* =0.02). Patients with LVI are more prone to LNM than those without (*p <*0.001). No significant difference was observed in incidence of LNM due to perineural invasion. The incidence of LNM was significantly higher in patients with CEA >5ng/ml (*p <*0.001). According to the results in **Figure 2**, we screened two monosaccharides: Gal and Man. And the high expressions of Gal and Man were associated with the incidence of LNM (*p <*0.001, *p <*0.001, respectively). There was no significant difference with respect to gender, age, BMI, and tumor location between patients with LNM and those without LNM. The results were shown in detailed in **Table 1**.

**Table 1:** Univariate analysis of risk factors for lymph node metastasis in colorectal cancer.

	n	Node negative (–)	Node positive (+)	*p* value
**Total**	252	130	122	
**Gender**				0.363
**female**	94	45	49	
**male**	158	85	73	
**Age**				0.307
**<60**	81	38	43	
**≥60**	171	92	79	
**BMI**				0.681
**≤28**	201	105	96	
**>28**	51	25	26	
**Location**				0.421
**colon**	103	50	53	
**rectum**	149	80	69	
**Tumor size**				0.027
**<2cm**	162	92	70	
**≥2cm**	90	38	52	
**Histologic grade**				<0.001
**G1, G2**	198	116	81	reference
**G3**	30	9	22	0.002
**mucin-producing subtype**	24	5	19	<0.001
**Depth of invasion**				0.02
**T1-T2**	48	32	16	
**T3-T4**	204	98	106	
**LVI**				<0.001
**yes**	102	30	72	
**no**	150	100	50	
**Perineural invasion**				0.112
**yes**	142	67	75	
**no**	110	63	47	
**CEA (ng/ml)**				<0.001
**≥ 5**	97	36	61	
**< 5**	155	94	61	
**Gal**				<0.001
**low (<cutoff)**	204	118	86	
**high (≥cutoff)**	48	12	36	
**Man**				<0.001
**low (<cutoff)**	167	101	59	
**high (≥cutoff)**	85	29	63	

The p value was calculated by Chi-square Test or Fisher exact test. Histologic grade: G1, well differentiated adenocarcinomas; G2, moderately differentiated adenocarcinomas; G3, poorly differentiated adenocarcinomas; mucin-producing subtype: signet ring cell type or mucinous carcinomas; LVI, lymphovascular invasion.

### Risk factors for LNM in CRC by univariate and multivariate analyses

The univariate analysis demonstrated that tumor size (≥ 2cm), histologic grade (G3), histologic grade (mucin-producing subtype), depth of invasion (T3-T4), LVI, high CEA, high Gal and high Man were associated with LNM. Subsequently, the stepwise logistic analysis showed that the independent risk factors for LNM in CRC were histologic grade (G3) (OR =2.60, *p* =0.043), histologic grade (mucin-producing subtype) (OR =3.38, *p* =0.032), LVI (2.42, *p <*0.01), CEA level(>0.5ng/ml) (OR =1.85, *p* =0.042) and Man (high) (OR =2.65, *p* =0.006). The results of multivariate analysis were listed in **Table 2**.

**Table 2:** Multivariate logistic regression analysis of lymph node metastasis in colorectal cancer.

	Odds ratio	95% CI	*p* value
**Tumor size (≥2cm)**	0.63	0.34-1.14	0.126
**Histologic grade**			0.020
**G3**	2.60	1.03-6.58	0.043
**mucin-producing subtype**	3.38	1.11-10.31	0.032
**Depth of invasion (T3-T4)**	1.25	0.59-2.68	0.56
**LVI**	2.42	1.28-4.57	<0.01
**CEA (≥ 5 ng/ml)**	1.85	1.02-3.34	0.042
**Gal (high)**	1.89	0.80-4.51	0.149
**Man (high)**	2.65	1.32-5.31	0.006

Histologic grade: G3, poorly differentiated adenocarcinomas; mucin-producing subtype, mucinous or signet ring cell type; LVI, lymphovascular invasion.

### The performance of Man and Gal in predicting the progonosis of CRC patients

**Figure 4:**
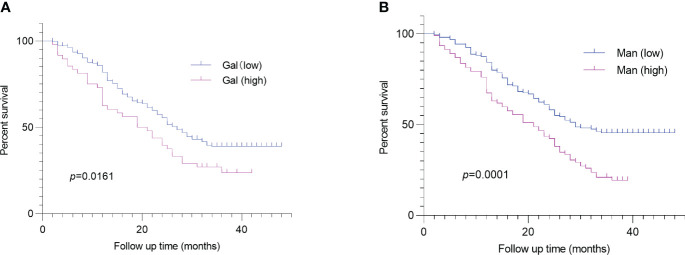
Log-rank analysis revealed that serum Gal **(A)** and Man **(B)** were associated with poor prognosis of CRC patients. CRC, colorectal cancer; Gal, galactose; Man, mannose.

we obtained the survival status of CRC patients included in this study through hospitalization records inquiries and telephone follow-up. Survival analysis revealed that serum Gal and Man were markedly related to poor prognosis of CRC patients (*p* =0.016, *p* =0.0001, respectively) (**Figures 4A, B**). In addition, we evaluated the performance of clinical pathological factors and serum monosaccharides in predicting the progonosis of CRC patients. After the univariate and multivariate Cox regression analysis, we demonstrated that histologic grade (G3) (HR 1.61; *p* =0.035), histologic grade (mucin-producing subtype) (HR 1.77; *p* =0.024), depth of invasion (T3-T4) (HR 1.86; *p* =0.01), LVI (HR 2.22; *p <*0.001), and high serum Man (HR 1.75; *p* =0.004) were independent risk factors for poor prognosis. The detailed information was shown in **Table 3**.

**Table 3:** Univariate and multivariate Cox-regression analysis of the factors affecting prognosis in colorectal cancer.

	Univariate			Multivariate		
	HR	95%CI	*p* value	HR	95%CI	*p* value
**Gender (female)**	1.22	0.88-1.69	0.214			
**Age (≥60)**	1.03	0.75-1.40	0.868			
**BMI (>28)**	0.76	0.52-1.10	0.172			
**Location (rectum)**	1.22	0.89-1.68	0.208			
**Tumor size (≥2cm)**	1.11	0.81-1.52	0.524			
**Histologic grade**			<0.001			0.017
**Histologic grade (G3)**	1.91	1.23-2.97	0.004	1.61	1.03-2.51	0.035
**Histologic grade (mucin-producing subtype)**	2.53	1.58-4.05	<0.001	1.77	1.08-2.89	0.024
**Depth of invasion (T3-T4)**	2.22	1.55-3.19	0.0005	1.86	1.16-3.00	0.01
**LVI**	2.21	1.54-3.20	<0.001	2.22	1.60-3.07	<0.001
**Perineural**	1.34	0.97-1.86	0.090			
**CEA (>5ng/ml)**	1.362	1.00-1.857	0.048	1.02	0.73-1.43	0.912
**Gal (high)**	1.56	1.02-2.39	0.016	0.89	0.57-1.38	0.603
**Man (high)**	1.89	1.35-2.64	<0.001	1.75	1.20-2.56	0.004

Histologic grade: G3, poorly differentiated adenocarcinomas; mucin-producing subtype, mucinous or signet ring cell type; LVI, lymphovascular invasion.

### Serum Man was elevated in early-stage CRC patients with LNM

**Figure 5:**
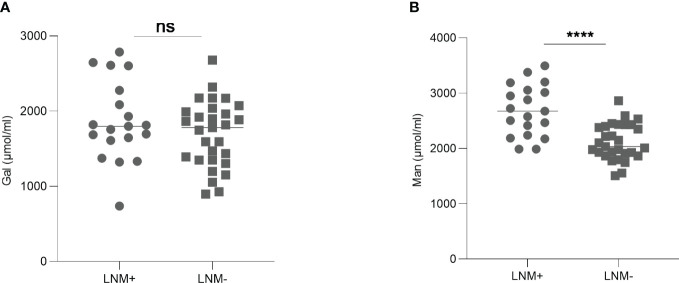
The levels of Gal **(A)** and Man **(B)** in early-stage CRC patients with or without LNM. Gal, galactose; Man, mannose; CRC, colorectal cancer; LNM, lymph node metastasis; ns, not significant; ****p ≤0.0001.

To investigate the predictive ability of serum monosaccharides for LNM in early-stage CRC patients, we compared the levels of Gal and Man in T1/T2-stage CRC patients with and without LNM. The results showed that Gal level was not significantly different between the two groups. However, serum Man level was higher in LNM-positive patients (*p <*0.0001) (**Figure 5**).

In addition, we compared the levels of CEA and Man between T1N0 and T1N1 patients. The result showed there was no significant difference in CEA levels between the two groups (*p* =0.410); However, compared with T1N0 patients, serum Man levels were elevated in T1N1 patients (*p* =0.026) (**Figure S1**).

### Association between serum monosaccharides and clinicopathologic parameters

The levels of Man and Gal were found to have no relationship with tumor location, size, and depth of invasion. Regarding histologic grade, the levels of Man were elevated in mucin-producting subtype (*p* =0.013). In addition, the levels of Man and Gal were not associated with parameters such as gender, age and BMI. However, in patients with high expression of CEA, the levels of Man (*p* =0.002) and Gal (*p* =0.018) were elevated. The detailed information was shown in **Table S1**.

**Figure 6:**
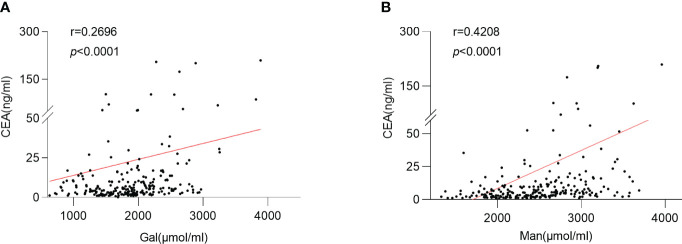
Relationship between serum monosaccharides and CEA. **(A)** Correlation between the serum Gal and CEA; **(B)** Correlation between the serum Man and CEA; Gal, galactose; Man, mannose; CEA, carcinoembryonic antigen.

The relationship was observed between serum Gal and CEA levels (*p <*0.0001; r =0.2626) (**Figure 6A**). And the serum Man level was positively correlated with the CEA level in CRC patients (*p <*0.0001; r =0.4208) (**Figure 6B**).

## Discussion

Glycosylation, in simple terms, is the enzymatic process that glycans attach to lipids or proteins (16). The variability of glycan structures gives them great ability to modulate the biological functions of glycoproteins (10). Glycans are known to act in cell adhesion, migration and intracellular signal transduction (17), and they are correlated with tumor invasion and metastasis (18). Alterations in protein glycosylation have been identified as hallmarks in tumorigenesis and progression (16). The glycan structure also altered during disease progression (19, 20), and some previous reports have demonstrated that glycans could be used as biomarkers in cancer diagnosis and metastasis (14, 21). However, it is difficult to detect glycans due to their abundant varieties and complex structures. It is well known that there are nine donors in mammals supplying polysaccharides for glycosylation, including Fuc, GalN, GlcN, Gal, Glc, Man, GlcA, Xyl and SA (9). Our research group has developed a high-throughput and easily generalized method to obtain circulating monosaccharides based on chromatography (8). To the best of our knowledge, this is the first study to demonstrate that the level of serum Man can be used as predictor of LNM and poor prognosis in CRC.

Our results showed that, among six serum monosaccharides, the Man level was elevated in LNM-positive CRC patients. Further, this study revealed that high serum Man was associated with adverse outcomes of CRC, and it was the independent risk factors for poor prognosis. The previous study found that abnormal high-mannose on tumor cell surface could enhance the capability of cell adhesion, further promoting tumor invasion and metastasis (13). Lyndsey et al. (22). found that the serum Man level in esophageal cancer patients was higher than that in the healthy, and it was higher in advanced patients than in early patients, which was consistent with our results. In previous studies, increased high-mannose glycans have been found in tumor tissues (21, 23), cell lines (24), and serum (14) of CRC patients. However, why high-mannose N-glycans elevated in cancer remains uncertain. This may be due to the accumulation of precursors caused by incomplete synthesis of N-glycans (25). Ganapati et al. found that aberrant α-mannosidase IA and glycosyltransferases lead to the formation of high-mannose glycans in prostate cancer cells (26). In addition, Fanny et al. indicated that in early-stage CRC patients, high-mannose N-glycans were correlated with malignant progression of disease, as it could promote cell proliferation (21). Mariana et al. (16). demonstrated that CRC cells could use the increased aberrant N-glycans to escape immune surveillance. Besides CRC, high-mannose N-glycan levels also elevated in papillary thyroid microcarcinoma (27), prostate cancer (28) and breast cancer (13, 25), and they were frequently increased in cancer metastasis.

Moreover, our results showed that serum Gal level was higher in CRC patients with LNM than in those without LNM. Xu et al. (29). found that Gal level was higher in CRC carcinomas than in normal control mucosa and precancerous lesions. However, Marcelo et al. (14). showed decreased galactosylation in serum of CRC patients. In addition, fucosylation level was altered in CRC (14, 24), but there was no difference in Fuc level between patients with LNM and those without LNM in our study. One possible explanation was that the serum degraded monosaccharides were affected by various glycans from tissues, cells, glycoproteins, etc. Therefore, the variations of partial glycosylation may not be consistent with changes in total monosaccharide levels. This still needs further exploration.

CEA is the most frequently used marker for CRC screening, diagnosis and monitoring. We compared the performance of CEA and serum monosaccharides in prediction of LNM. The ROC curve analysis showed that the predictive performance of Man for LNM was better than CEA. In our study, we also found that the serum Man level in CRC patients was positively correlated with CEA. As a glycoprotein, the glycan chains of CEA were variable, which were mainly composed of Man, Fuc, SA, Gal and N-acetylglucosamine (30). Zhao et al. (31). demonstrated stage-dependent alterations of CEA glycosylation patterns in CRC. In specifically, mannose level increased in tumor-associated CEA.

With regard to the analysis of clinicopathologic factors, the results of this study suggested that independent risk factors for LNM in CRC included poorer histologic grade, LVI and CEA; in addition, histologic grade (G3), histologic grade (mucin-producing subtype), depth of invasion(T3-T4), and LVI were associated with poor prognosis. The above results were consistent with previous studies (32–35). The consensus molecular subtypes (CMS) of colorectal cancer indicated that the metabolic subtype (CMS3) potentially associated with Man metabolism and mucinous differentiation (36). Therefore, the poorly differentiated tumors were separated into distinct categories: G3 and mucin-producing subtypes. In this study, the levels of serum Man were elevated in CRC patients with mucin-producing subtype, which is consistent with previous research (36).

In the latest CRC treatment guidelines (5), the JSCCR noted that indication for endoscopic resection was intramucosal carcinoma or slight submucosal carcinoma with little possibility of LNM. Therefore, the evaluation of LNM in early CRC is valuable to guide clinical treatment. Our results showed that serum Man level was higher in early CRC patients with LNM. In addition, compared to T1N0 patients, the levels of Man were elevated in T1N1 patients, while no significant difference was observed in CEA levels between the two groups. This suggested that Man would be a promising marker for predicting LNM in early-stage CRC. However, due to the small sample size of early-stage patients, the analysis is not detailed. Subsequent accurate stratified analysis will be necessary in the future. Furthermore, no external validation was conducted in this study. Nevertheless, the samples we collected were screened using rigorous inclusion and exclusion criteria, suggesting that our findings might be reliable and reproducible.

## Conclusions

The Man is superior to CEA in LNM prediction for CRC patients, and it is the independent risk factor for LNM. Serum Man level was associated with poor prognosis of patients with CRC. Man is expected to be a marker for LNM and prognosis in CRC patients.

## Data availability statement

The original contributions presented in the study are included in the article/**Supplementary Material**. Further inquiries can be directed to the corresponding authors.

## Ethics statement

The studies involving humans were approved by the Ethics Committee of the Affiliated Hospital of Qingdao University. The studies were conducted in accordance with the local legislation and institutional requirements. Written informed consent for participation in this study was provided by the participants’ legal guardians/next of kin. Written informed consent was obtained from the individual(s) for the publication of any potentially identifiable images or data included in this article.

## Author contributions

XW: participated in the research design, conducted experiments, and drafted the paper. HL: involved in clinical data collection. XC: designed and supervised the study. ZT: designed, supervised thes study, and wrote the paper. All authors contributed to the article and approved the submitted version.
