# Supplementary material for: High serum mannose in colorectal cancer: a novel biomarker of lymph node metastasis and poor prognosis
Source: Front Oncol. 2023 Aug 22;13:1213952. doi: 10.3389/fonc.2023.1213952 (PMC10479890; doi:10.3389/fonc.2023.1213952)
Supplement: Supplementary file 1 [file Presentation_1.pdf]

Supplementary Material

1. Figure

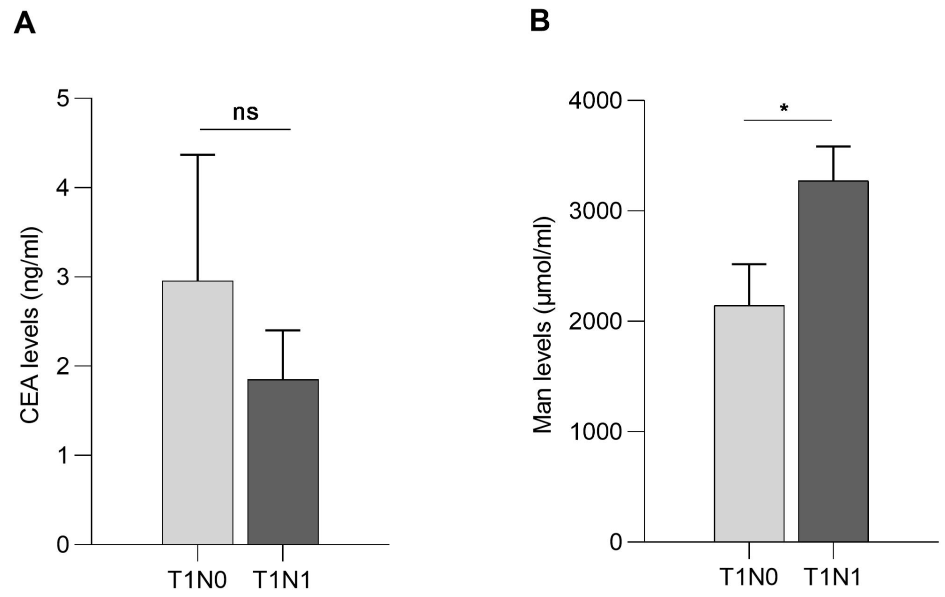

Figure S1. The levels of CEA **(A)** and Man **(B)** between T1N0 and T1N1 CRC patients. CEA, carcinoembryonic antigen; Man, mannose; CRC, colorectal cancer; ns: not significant, *: $p \leq 0.05$.

2. Table

Table S1. The association between serum monosaccharides and clinicopathologic parameters.

	n	Gal	*P* value	Man	*P* value
**Gender**			0.108		0.433
**male**	158	1824 ± 552.6		2473 ± 494.2	
**female**	94	1940 ± 550.6		2525 ± 539.4	
**Age**			0.060		0.246
**<60**	81	1963 ± 567.2		2546 ± 537.4	
**≥60**	171	1822 ± 543.0		2466 ± 497.7	
**BMI**			0.949		0.673
**≤28**	201	1866 ± 565.5		2499 ± 498.7	
**>28**	51	1872 ± 509.7		2465 ± 561.8	
**Location**			0.259		0.829
**colon**	103	1915 ± 421.2		2484 ± 469.7	
**rectum**	149	1835 ± 628.4		2498 ± 539.4	
**Histologic grade**			0.219		0.008
**G1, G2**	198	1838 ± 555.1	reference	2443 ± 513.1	reference
**G3**	30	2018 ± 446.0	0.220	2604 ± 417.0	0.232
**mucin-producing subtype**	24	1924 ± 644.0	0.751	2775 ± 516.8	0.013
**Tumor size**			0.869		0.059
**<2cm**	162	1872 ± 565.8		2447 ± 516.7	
**≥2cm**	90	1860 ± 534.1		2574 ± 493.3	
**Depth of invasion**			0.159		0.059
**T1-T2**	48	1766 ± 480.7		2367 ± 483.5	
**T3-T4**	204	1891 ± 567.9		2522 ± 514.1	
**CEA (ng/ml)**			0.018		0.002
**≥5**	97	1971 ± 625.6		2618 ± 534.8	
**<5**	155	1802 ± 494.5		2413 ± 481.0	

Histologic grade: G1, well differentiated adenocarcinomas; G2, moderately differentiated adenocarcinomas; G3, poorly differentiated adenocarcinomas; mucin-producing subtype: signet ring cell type or mucinous carcinomas.
